# Late Complications following Endoscopic Sphincterotomy for Choledocholithiasis: A Swedish Population-Based Study

**DOI:** 10.1155/2014/745790

**Published:** 2014-10-16

**Authors:** A. Langerth, L. Brandt, A. Ekbom, B.-M. Karlson

**Affiliations:** ^1^Department of Surgery, University Hospital, 75185 Uppsala, Sweden; ^2^Department of Medical Epidemiology and Biostatistics, Karolinska Institute, 171 77 Stockholm, Sweden

## Abstract

In order to assess the risk of long-term complications following endoscopic sphincterotomy (ES) for common bile duct stones (CBDS), we conducted a cohort study. The study included 1,113 patients who underwent ES for CBDS in six different hospitals in central Sweden between 1977 and 1990. Through the use of the Swedish population registry, each patient was assigned five population-based controls matched for sex and age. Linkage to the Inpatient Registry yielded information on morbidity and mortality for the patients as well as for the controls. After one year of washout, there were 964 patients available for follow-up. The mean age was 70.6 years, 57% were women, and the mean length of follow-up was 8.9 years. The patients' overall morbidity was significantly higher and we observed a tendency towards increased mortality as well. Recurrent CBDS was diagnosed in 4.1% of the patients. Acute cholangitis with a hazard ratio (HR) of 36 (95%CI 11–119.4) was associated with recurrent CBDS in 39% of the patients. HR for acute pancreatitis was 6.2 (95%CI 3.4–11.3) and only one patient had CBDS at the same time. In conclusion, we consider acute pancreatitis and cholangitis both as probable long-term complications after ES.

## 1. Introduction

Long-term results after endoscopic sphincterotomy (ES) have been the topic of several studies, with a complication rate, including recurrent common bile duct stones (CBDS), between 5 and 24%. Most authors, however, report frequencies around 10% [1–5]. There is currently no agreed definition of a late complication, though, and various publications differ greatly.

As the summary in Table 1 shows, the rate of recurrent CBDS varies between 3.5 and 14% [1–11]. Keizman et al. [12] demonstrated recurrent CBDS in the elderly (older than 79) and in younger patients (less than 51 years old) of 20% and 4%, respectively. The largest study so far of 7, 585 patients by Seifert et al. in 1982 [13] found recurrent stones in about 5.8%, but the follow-up time was not reported. Some of the studies include reports of acute cholangitis together with CBDS, but it was not until 1996 that Prat et al. [7] described “sine materia cholangitis.” The mean follow-up was 9.6 years and three out of 154 patients (1.9%) developed acute cholangitis without recurrent CBDS. In a more recent study by Costamagna et al. [4], with 458 patients and a mean follow-up of 6.8 years, four of the 458 patients (0.9%) had a diagnosis “sine materia cholangitis.” In contrast, some authors report no cases of acute cholangitis without concurrent CBDS [1, 2, 9]. Few publications have explored that issue.

In order to further assess the risk of long-term complications following ES for CBDS, we conducted a cohort study to assess complication rates and total morbidity as compared to the background population.

## 2. Materials and Methods

A total of 1, 113 patients were identified from local registers in six hospitals in central Sweden between 1977 and 1990. This cohort included all patients who underwent ES due to stones in the CBD, while patients with suspected malignant strictures were excluded. By using the Swedish population registry, we identified five controls, who were alive on January 1, 1977, from the background population for each case, matched by sex and age. All patients and controls were identified by their national registration numbers, unique for each resident in Sweden [14], and record linkages were made to the Inpatient Registry, which contains information on all public inpatient treatment in Sweden. Since there is almost no private inpatient treatment in Sweden, with patients obliged to use the public hospitals located in their county of residence, the Inpatient Registry is essentially population-based. All patients were followed until December 31, 1999, or until the patient's death or that of its last control, as at least one control for each patient has to be alive. In the analyses, the first year after the procedure was excluded in the follow-up, resulting in the exclusion of 127 patients due to their death within the first year. In addition, 21 patients were excluded as all their controls were dead before the time for the patients ES and one patient emigrated within the first year. Thus, the study consisted of 964 patients (57% women) available for follow-up. The mean age of the patients was 70.6 years and the mean length of follow-up was 8.9 years for the patients and 8.3 years for the controls.  Table 2 presents this material. By linking to the Inpatient Registry we were able to retrieve information on total morbidity, morbidity with an underlying diagnosis such as cholecystitis, recurrent stones in the CBD, cholangitis, pancreatitis, jaundice, and advanced alcoholism, and mortality. Total morbidity is defined as all sicknesses requiring inpatient care. We have no information as to whether a prior cholecystectomy or ES had been performed as the linkage to the Inpatient Registry was prospective, containing just the information after the performance of ES.

## 3. Statistics

Multivariate analysis for total morbidity, local morbidity, and mortality was performed taking into account age and sex using the Cox proportional hazards regression model [15].

## 4. Results

During the first year, mortality was 11.5% (127/1112) for the patients and 4.4% (194/4396) for the controls. After exclusion of the first year, the overall morbidity as well as mortality after ES was significantly increased as compared to the background population.

We found a significantly higher mortality after ES in both men (HR 1.29 (95%CI 1.05–1.59)) and women (HR 1.30 (95%CI 1.06–1.60)) in the 65–79 age group and for men younger than 50 (HR 3.22 (95%CI 1.22–8.48)) compared to the background population (Table 3). The overall morbidity showed higher health care consumption 3-4 years after ES in all age groups and both genders except for men older than 80 (Table 4), although advanced alcohol abuse was found to be higher in the background population.

**Figure 1:**
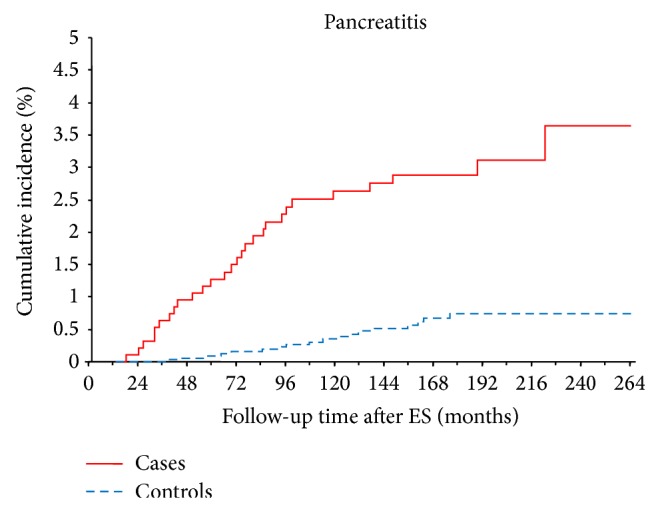
Cumulative incidence of pancreatitis 12 months and more after ES.

Recurrent bile duct stones were identified in 40 (4.1%) of the patients with an HR of 8.9 (95%CI 5–15.7) (Tables 5 and 6). Strictures of the common bile duct were found in 9 (0.9%) of the 964 patients as compared to 4 (0.1%) of the 3 811 controls (HR = 13.4 (95%CI 3.6–49.6)). Twenty-eight (2.9%) patients developed acute cholangitis (HR 36 (95%CI 11–11.9)) and 11 with and 17 without concomitant bile duct stones (Tables 5 and 6). Most of the patients with cholangitis were seen during the first four years following ES. Of the 3, 811 controls, there were three (0.001%) with acute cholangitis during the whole observation time. Acute pancreatitis was diagnosed in 26 (2.7%) of the patients, but only one of them had concomitant CBDS (Table 6). The HR for pancreatitis after ES was 6.2 (95%CI 3.4–11.3). Late complications in the form of acute pancreatitis were diagnosed over the whole observation time, but most cases were seen during the first 8 years after ES (Figure 1).

## 5. Discussion

This prospective cohort study shows an increased risk for pancreatitis and cholangitis after ES for CBDS without recurrence of CBDS. Furthermore, the overall morbidity as well as mortality after ES was significantly increased as compared to the background population.

In previously published reports, the time between ES and the start of follow-up differs. Ando et al. in 2003 [11] and Bergman et al. in 1996 [6] reported recurrent stones in 11.3% and 14%, respectively. They both defined long-term follow-up as more than 30 days after ES. In 1988, Ikeda et al. [16] showed recurrent stones in 5.8% of the patients, 6 months or more after ES.

In our study, we have a washout period of 365 days and no episodes of inpatient care during this time were assessed. The higher mortality of our patients (11.5%) compared to that of the background population (4.4%) during the first year after the procedure was confined to the first 30 days after the procedure and was probably due to a combination of direct complications from the procedure as well as a selection of patients in poor condition.

After the first year, there was a higher mortality in both men and women in the 65–80 age group but not among those older than 80. This is probably due to selection bias; that is, those subjected to ES are healthier than the normal population within this age group. However, it is more difficult to explain the higher mortality in men younger than 50, but this could be due to the selection of patients with comorbidity. This could be the reason why the younger patients are not subjected to surgery.

Of the 28 patients with acute cholangitis, only 11 (39%) had concurrent choledocholithiasis. Thus, there are as many as 17 patients (61%) without a clear underlying cause for cholangitis. One explanation could be that this is a long-term complication following ES. This has previously been described by Uchiyama et al. [17] and that was why many institutions preferred to perform endoscopic papillary balloon dilatation (EPBD) instead of ES, especially in younger patients. Unfortunately, according to the latest review [18], EPBD is associated with a higher incidence of postoperative pancreatitis and has to be pursued with less hazard techniques. In contrast to the previous discussion, Tanaka et al. [9] could not identify any case from the 410 patients studied who had cholangitis without recurrent bile duct stone. However, few authors have commented whether there have been cases of cholangitis without concomitant bile duct stones or not.

Most interesting is the significantly higher incidence of pancreatitis for those patients without concurrent CBDS. Several authors [19, 20] have reported results that indicate reflux of duodenal contents up in the bile and pancreatic duct after ES but no obvious unfavourable effects. However, this is still a subject of great interest and importance in the long-term follow-up after ES, as the link between inflammation and cancer is well-known nowadays [21]. Pancreatitis due to such reflux, as a long-term outcome after ES, can be the explanation of our results, concerning the high incidence of pancreatitis without concurrent CBDS.

To the best of our knowledge, the higher long-term risk of pancreatitis after ES has never been described in the literature until now. In addition, alcohol abuse was less common in the patient group compared to the background population, which might be due to a decreased diagnostic intensity in patients with known gallstones during the inpatient care for pancreatitis, but probably not during other inpatient care periods. Moreover, alcohol has been shown to be associated with a lower prevalence of gallstones disease [22].

The strength of this study is its prospective and population-based design and, to our knowledge, it is the only cohort study made on this subject. Furthermore, we can present a mean follow-up period of more than 8 years.

Lack of data is one limitation in this study as we only have information about inpatient care concerning diagnosis and surgical procedures after ES. For example, cholecystectomy before ES is not noted and thus remains unknown. The entire patient data was made anonymous after the statistic process and the medical record was not accessible.

Alcohol abuse information is another problem, since this is probably underreported in the Inpatient Registry with only the most serious alcohol abuse being registered.

In conclusion, we consider acute pancreatitis and acute cholangitis both as probable long-term complications after ES. However, additional studies are needed in order to establish a causal association.

## Figures and Tables

**Table 1:** Summary of some previously published follow-up data.

Year	Study	Number of patients	Mean length of follow-up (years)	Mean age	Recurrent CBDS (%)	Total complications (%)
1996	Bergman et al. [6]	94	14	51	14	24
	Prat et al. [7]	156	9.7	55	3.5	5.8

1997	Wojtun et al. [1]	324	6.0	58.8	5.6	9.9

1998	Pereira-Lima et al. [8]	201	6.2	67.9	8.0	15.4
	Sugiyama and Atomi [2]	103	14.2	50	3.9	9.7
	Tanaka et al. [9]	410	10.2	64	10.7	12.3

2000	Saito et al. [10]	371	7.7	65.4	9.7	20

2002	Schreurs et al. [3]	310	6.2	69	7.4	10
	Costamagna et al. [4]	458	6.8	63	9.2	11.1
	Sugiyama and Atomi [5]	135	14.8	49	8.9	11.9

2003	Ando et al. [11]	983	np	np	11.3	np

**Table 2:** Patient distribution correlated to age, sex, and start of follow-up.

		Patients	% of the total cohort
Gender	Women	546	57
	Men	418	43

Age at the start of follow-up (years)	<50	80	8
	50–64	200	21
	65–79	397	41
	80+	287	30

Calendar year at the start of follow-up	1977–80	74	8
	1981–85	551	57
	1986–91	337	35

		Median/mean	Range

Age of the patients at start of follow-up		73/70.6	21–95

Number of years in follow-up	Patients	8.9/8.9	0–20.9
	Controls	7.4/8.3	0–20.9

**Table 3:** The hazard ratio (HR) of mortality after ES for the cohort over the entire follow-up period and for the first five years divided by age and sex.

Sex	Age (years)	Total time	First five years
		HR (95%CI)	HR (95%CI)
M	<50	3.22 (1.22–8.48)	10.0 (0.91–110.27)
	60–64	1.29 (0.87–1.92)	0.82 (0.34–1.96)
	65–79	1.29 (1.05–1.59)	1.49 (1.09–2.03)
	80+	0.81 (0.61–1.06)	0.77 (0.56–1.06)

F	<50	1.48 (0.48–4.53)	2.50 (0.41–15.06)
	60–64	1.38 (0.92–2.07)	1.75 (0.78–3.93)
	65–79	1.30 (1.06–1.60)	2.01 (1.45–2.77)
	80+	1.02 (0.82–1.27)	0.81 (0.61–1.09)

**Table 4:** The hazard ratio of morbidity for the cohort divided by age, sex, and time after ES.

Sex	Age (years)	1-2 years after ES HR (95%CI)	3-4 years after ES HR (95%CI)	>5 years after ES HR (95%CI)
M	<50	3.6 (1.7–7.5)	3.5 (1.0–12.2)	4.0 (1.9–8.5)
	50–64	1.5 (1.0–2.2)	2.7 (1.6–4.6)	1.9 (1.2–3.1)
	65–79	1.9 (1.5–2.6)	2.1 (1.3–3.4)	2.0 (1.2–3.4)
	80+	1.4 (1.0-2.0)	2.3 (0.7–7.6)	

F	<50	1.8 (1.0–3.1)	3.5 (1.4–8.8)	2.1 (1.2–3.6)
	50–64	2.0 (1.4–2.9)	3.2 (1.9–5.4)	2.2 (1.5–3.1)
	65–79	2.0 (1.6–2.5)	2.1 (1.3–3.4)	1.8 (1.2–2.5)
	80+	1.3 (1.0–1.8)	2.5 (1.3–4.8)	2.2 (0.9–5.4)

**Table 5:** Hazard ratio (HR) of local morbidity after ES for the entire follow-up time.

	HR (95%CI)	*P* value
Acute cholangitis	36.2 (11–119.4)	<0.001
Acute pancreatitis	6.2 (3.4–11.3)	<0.001
CBDS	8.9 (5–15.7)	<0.001

**Table 6:** Local morbidity for the patients in the whole follow-up time.

	Total number of patients	Patients with single diagnosis	Patients with several diagnosis	Cholangitis	CBDS	Jaundice	Acute pancreatitis
Cholangitis	28	15	13	—	11	3	1
CBDS	40	27	13	11	—	3	1
Jaundice	8	4	4	3	3	—	0
Acute pancreatitis	26	24	2	1	1	0	—
